# Smokers' recall of Australian graphic cigarette packet warnings & awareness of associated health effects, 2005-2008

**DOI:** 10.1186/1471-2458-11-238

**Published:** 2011-04-17

**Authors:** Caroline L Miller, Pascale G Quester, David J Hill, Janet E Hiller

**Affiliations:** 1 Cancer Council SA, 202 Greenhill Rd, Eastwood, South Australia, Australia; 2 Discipline of Public Health, School of Population Health and Clinical Practice, University of Adelaide, South Australia, Australia; 3 University of Adelaide Business School, South Australia, Australia; 4 The Cancer Council Victoria, 1 Rathdowne St, Carlton, Victoria, Australia; 5 Australian Catholic University, Melbourne, Australia

## Abstract

**Background:**

In 2006, Australia introduced graphic cigarette packet warnings. The new warnings include one of 14 pictures, many depicting tobacco-related pathology. The warnings were introduced in two sets; Set A in March and Set B from November. This study explores their impact on smokers' beliefs about smoking related illnesses. This study also examines the varying impact of different warnings, to see whether warnings with visceral images have greater impact on smokers' beliefs than other images.

**Methods:**

Representative samples of South Australian smokers were interviewed in four independent cross-sectional omnibus surveys; in 2005 (n = 504), 2006 (n = 525), 2007 (n = 414) and 2008 (n = 464).

**Results:**

Unprompted recall of new graphic cigarette warnings was high in the months following their introduction, demonstrating that smokers' had been exposed to them. Smokers also demonstrated an increase in awareness about smoking-related diseases specific to the warning messages. Warnings that conveyed new information and had emotive images demonstrated greater impact on recall and smokers' beliefs than more familiar information and less emotive images.

**Conclusions:**

Overall graphic pack warnings have had the intended impact on smokers. Some have greater impact than others. The implications for policy makers in countries introducing similar warnings are that fresh messaging and visceral images have the greatest impact.

## Background

The World Health Organization's Framework Convention on Tobacco Control (FCTC) is a global health treaty designed to help curb the global tobacco epidemic and associated burden of disease and mortality [1]. Countries that ratify the FCTC commit themselves to a schedule of tobacco control legislative reform in an effort to advance disease prevention and health promotion. The regulation of packaging and labelling of tobacco products is one component of a comprehensive approach (see Articles 6-14). Australia was one of the first 40 countries to ratify the FCTC, and so became a full Party on 27 February 2005. In early 2006, Australia followed Canada, Brazil, Singapore, Thailand, Venezuela and Panama in introducing new graphic cigarette packet warnings [2]. Many other countries have since introduced them or are in the process of doing so.

Cigarette packet warnings are an important form of health communication to consumers. Australia's graphic health warnings were designed to provide "a strong and confronting message to smokers about the harmful health consequences of tobacco products and convey the 'quit' message every time a person reaches for a cigarette" [3]. The stated intention was that graphic images would increase consumer awareness of the health effects of smoking, which would in turn decrease likelihood of smoking [3].

Theories of consumer behaviour and social psychology predict that a number of predisposing variables influence behaviour and the probability of behavioural change, with people's beliefs being an important contributor [4-7]. Consumer behaviour theory holds that behaviour change, such as stopping smoking, can be induced by increasing consumer perception that the behaviour is a 'problem' for them, requiring behavioural modification [4]. By increasing a person's belief that smoking leads to negative health consequences, pack warnings could change the consumer's satisfaction with his/her current status as a smoker and induce (or increase) his/her desire to quit, increasing the chances that s/he would try to quit.

It has been widely demonstrated that beliefs which are 'top of mind' for people or salient are also more likely to influence behaviour [5,8]. Hence, if pack warnings increase a person's awareness that smoking leads to particular negative health consequences, and the beliefs about those health consequence are salient for the smoker, they would be more likely to influence quitting behaviour.

Of course, other factors can also induce behavioural change such as other internal factors [6] and social and environmental factors also influence smoking behaviour [5]. Beliefs are, however, an important antecedent of behaviour change, and one that has the potential to be influenced by information contained in graphic cigarette packet warnings.

In order to change beliefs, consumer information first has to be noticed and attended to. Tobacco health warnings have also been shown to be effective in attracting and maintaining attention, as well as assisting information processing, provided the messages are clear, noticeable, strong, direct and frequently rotated [9]. International studies have demonstrated greater knowledge about particular health effects in countries where those health effects are the subject of a cigarette packet warning than in countries where they are not [10]. These studies have confirmed that smaller text-based cigarette packet warnings have lesser impact while larger warnings, including those with clear, simple language and graphic images, are associated with: better knowledge; higher recall; greater motivation to quit; and quit attempts [10-15]. Some smokers also take steps to avoid stronger warnings, particularly some graphic warnings [14]. Borland et al. [16] found no evidence that warning avoidance, arguably a defensive reaction against fear-arousing warnings, had a negative effect on quitting behaviour.

The new Australian graphic cigarette pack warnings (available for view elsewhere [17]) are larger than ever seen before on Australian cigarette packets and cover 30% of the front and 90% of the back of the pack. The graphic image of a health effect contrasts with the otherwise appealing aesthetics of the rest of the cigarette packaging. The Quitline number is 'stamped' on top of the graphic image on the backs of packs.

There are 14 different warnings divided into two sets; Set A and Set B [3]. The sets of warnings are rotated 12-monthly, including a 4 month transition period, during which any of the warnings from either set may appear. Set A only could appear on packs manufactured or imported from 1 March-31 October 2006. Set B only could appear on packs manufactured or imported from 1 March-31 October 2007.

The packs include a combination of new and familiar images and messages. Some messages had been on text-based packets for some time; others had not. Some images and messages had been used before in televised anti-tobacco social marketing campaigns; others had not. Table 1 lists the new warnings and the extent to which the text and imagery is new to Australian smokers. For example, "Smoking causes peripheral vascular disease", and "Smoking causes mouth and throat cancer" were unique in that they contained both new images and new messages and had not previously been the subject of text-based pack warnings or social marketing campaigns. Hence, these warnings would be novel for many smokers. By contrast "Smoking causes lung cancer" was introduced as a text-based pack warning in 1987 and the image on the packet was used in a televised anti-tobacco campaign from 1997.

**Table 1:** New cigarette packet warnings and previous use of warning components in Australia

Text	Image	First use of warning components		Previous TV anti-smoking campaign on health effect
		
		Text	Image	
Set A				
Smoking causes peripheral vascular disease	Gangrenous foot	2006 (Mar)	2006 (Mar)	No
Smoking causes emphysema	Dissected lung	2006 (Mar)	1997	Yes
Smoking causes mouth and throat cancer	Cancerous lip	2006 (Mar)	2006 (Mar)	No
Smoking clogs your arteries	Dissected artery	2006 (Mar)	1997	Yes
Don't let children breathe your smoke	Child on oxygen	2006 (Mar)	2006 (Mar)	Yes
Smoking - a leading cause of death	Bar chart	2006 (Mar)	2006 (Mar)	Yes
Quitting will improve your health	Quitline caller	2006 (Mar)	1998*	Yes
Set B				
Smoking causes blindness	Eye close up	2006 (Nov)	2000	Yes
Smoking doubles your risk of stroke	Dissected brain	2006 (Nov)	1998	Yes
Tobacco smoke is toxic	Beaker of chemicals	2006 (Nov)	2000*	Yes
Smoking harms unborn babies/(Smoking while pregnant may harm the unborn child)	Premature baby	2006 (Nov)/1995	2006 (Nov)	No
Smoking is addictive	Stained fingers	1995	2006 (Nov)	Yes
Smoking causes lung cancer	Tumour close up	1987	1997	Yes
Smoking causes heart disease	Heart surgery	1987	2006 (Nov)	Yes

* Essentially equivalent image to that was used in television campaign

We wanted to explore the changes in recall of the new warnings over time as well as changes in beliefs about the health effects of smoking, associated with the new system of graphic warnings. We also looked at the differential impact of individual new health warnings on smokers, given that the extent to which each of the new warnings: captures attention; delivers new information (or old information in new ways); is comprehended; changes awareness or beliefs about health effects; and is recalled, are all important aspects of information processing. These variables influence the degree to which different warnings may influence behaviour change.

One study has already indicated that Australian warnings were noticed by the majority of adolescents and led to increased cognitive processing about the health risks covered [18]. Another study demonstrated that new Australian health warnings were read and noticed more than UK's text only warnings and that they stimulated thoughts about the harms of smoking, thoughts about quitting and the behaviour of foregoing cigarettes [19]. Our study measured changes in smokers' basic beliefs about the different harms of smoking, at the adult population level over time, as the various warnings were rolled out. The study also measured degree of recall of specific warnings. In this study, changes in beliefs and recall were measured across smokers in the community as a whole and among different subgroups, such as younger smokers. The purpose of these sub-group analyses was to ascertain whether graphic cigarette packet warnings had differential impact with different demographic groups of smokers or whether any impact was universal. Anti-tobacco television campaigns have consistently demonstrated that images and messages eliciting a visceral response and messages that are novel or "new news" are more likely to be attended to and have impact on quitting behaviour [20-22]. Hence, it is hypothesized that new packet warnings which are most novel or contain the newest 'news' for smokers will result in the greatest attention to the pack warnings themselves, greatest recall of warnings and greatest increases in basic beliefs about smoking related illnesses. It is further hypothesized that visceral images will have greater impact on these variables than other images.

Other factors likely to influence behaviour change, including perceived ability to change behaviour, social and environmental factors are beyond the scope of the current study.

## Methods

### Sample

Data were collected as a part of the South Australian Health Omnibus Surveys; annual independent cross-sectional surveys of the South Australian population, undertaken from September to November. These population surveys involve a multistage, systematic, clustered area sample of households, with Australian Bureau of Statistics Collector's Districts as the sampling frame. Greater details on sampling are provided elsewhere [23]. At each selected household, one person aged 15 years or older whose birthday was due next was selected for interview. Structured interviews were conducted in the respondents' own homes by trained interviewers. Up to six call-back visits were made to each household in an attempt to obtain an interview if the respondent was not home.

The South Australian Health Omnibus Survey tool used the same methods each year. Data were weighted by household size, age, gender and local government area, so that estimates would reflect the South Australian population. Hence, the samples are directly comparable from year to year. Studies measuring changes over time in behaviour and attitudes in the South Australian population, using this tool and its comparable samples, have been accepted in many areas of inquiry [24-28].

Data for this study were collected in the South Australian Health Omnibus Surveys of 2005, 2006, 2007 and 2008. The survey achieved response rates of 70.9%, yielding 3047 interviews in total and 571 smokers in 2005; 63.8% with N = 2969 (609 smokers) in 2006; 62.7% with N = 2401 (478 smokers) in 2007; and 53.6% with N = 2824 (553 smokers) in 2008. Despite different response rates the samples from the four survey years did not differ significantly in age, gender or quitting experience. Respondents were classified as smokers if in response to the question: "Do you currently smoke: daily; at least weekly (but not daily); less often than weekly; or not at all", they answered other than "not at all". Similarly, respondents were classified as smokers of manufactured cigarettes according to their responses to the question "How often do you smoke manufactured cigarettes: Daily, weekly; less than weekly; or not at all". This study was restricted to the responses of smokers of manufactured cigarettes. Non-smokers (never-smokers and ex-smokers) were not included in this study because it was not expected that they would be exposed to or attuned to cigarette packet warnings.

The 2005 survey occurred before any new packet warnings were introduced, the 2006 survey occurred after Set A warnings were introduced and became prevalent in stores [29] but before Set B warnings were rolled out. The 2007 survey occurred after Set B warnings were introduced.

### Measurements

Participants were asked a series of questions. To measure top of mind awareness of the effects of smoking, participants were first asked "Which illnesses are caused by smoking?" Participants were not prompted with response options. Some but not all of the pre-coded response options matched the new warnings, as listed in Table 2. To assess recall of pack warnings, participants were asked "In the past 6 months, how often, if at all, have you noticed advertising or information that talks about the dangers of smoking, or encourages quitting". Prompted response options were "never", "rarely", "sometimes", "often" or "very often". If they did not respond "never" they were then asked "Where did you see that information?" Unprompted pre-coded responses included "TV", "radio", "cigarette packets", "cinema" and "internet". Smokers and ex-smokers were later asked "As far as you know, what do the warnings on cigarette packets say?" Pre-coded options for the unprompted responses included all new and previous cigarette packet warnings as well as "Quitline number", "Pictures of effects of smoking" and "Don't know/can't remember". Participants were also asked "Can you tell me the name of any services or programs available to help people quit smoking". Unprompted response options were "Quitline", "Quit campaign", "Nicotine Replacement Therapy", "Zyban/buproprion", "Talking to a doctor", "Alternative Therapy", "Other" and "Don't know". Subsequent to that, smokers were asked whether "During the past year, have you done any of the following: "Called the Quitline", and so on for other quitting services. All of these questions have been routinely used in the South Australian Health Omnibus Survey for 10 years.

**Table 2:** Awareness of health effects, Quitline and use of Quitline (unprompted) (smokers of manufactured cigarettes only)

		2005 (n = 504)	2006 (n = 525)	2007 (n = 414)	2008 (n = 464)
**Beliefs that smoking causes illness and/or damage to the body**					
Set A related beliefs	Text/Image				
Emphysema	New/Old	60%	**59%**	57%	52%
Mouth cancer	New/New	10%	**24% **^**a**^	21% ^a^	21% ^a^
Throat cancer	New/New	14%	**17%**	17%	22% ^b^
Gangrene	New/New	4%	**27% **^**a**^	25% ^a^	28% ^a^
Blocked arteries	New/Old	10%	**19% **^**b**^	14%	12% ^β^
					
Set B related beliefs					
Blindness/Eye damage	New/Old	16%	11%	**25% **^**b**^	16% ^ϕ^
Stroke	New/Old	9%	8%	**17% **^**b**^	11% ^ϕ^
Harms unborn babies*	New/Old	8%	5%	**13% **^**c**^	13%
Addiction^#^	Old/New	7%	10%	**10%**	10%
Heart disease^#^	Old/New	39%	34%	**36%**	33%
Lung cancer^#^	Old/New	55%	53%	**55%**	55%
					
'Control' beliefs					
Asthma	n/a	20%	19%	15%	6% ^a^
Cough	n/a	9%	6%	8%	12%
Blood pressure	n/a	11%	7%	7%	7%
Impotence	n/a	0%	0%	<1%	<1%
					
**What services are available to help smokers quit**					
Quitline		71%	75%	81% ^a^	78% ^**c**^
					
**Correct recall of Quitline number**		5%	n/a	14% ^b^	n/a
					
**Method of quit attempt **(of those who tried to quit in the past year)		(n = 201)	(n = 209)	(n = 163)	(n = 164)
Called the Quitline		7%	8% ^b^	11%	12%

* Similar to previous warning "smoking in pregnancy may harm the unborn child" ^# ^Old warnings and new warning

^a^Significant difference from baseline (2005) p < 0.001

^b^Significant difference from baseline (2005) p < 0.01

^c^Significant difference from baseline (2005) p < 0.05

^β^Significant difference from 2006 p < 0.01

^ϕ^Significant difference from 2007 p < 0.05

Newness or novelty of text and images included in the graphic cigarette packet warnings is defined by their use in previous population based tobacco control interventions, namely text-based cigarette packet warnings and mass media cessation campaigns. Table 1 provides information about previous use of pack warnings text content and images. When text has been used previously in text-based cigarette packet warnings it is classified as "old". When text has not been used previously in text-based cigarette packet warnings it is classified as "new". When images have been used in mass media campaigns previously they are classified as "old" and when they have not they are classified as "new".

### Statistical Analyses

Data analyses were undertaken using STATA v10.0. STATA provides survey estimating tools required to account for this survey design. The survey estimating tools adjust the standard errors to account for the design which involved clustering by Australian Census District, stratification (metropolitan vs. rural) and data that are weighted to the population. Inter-year and intra-year differences between proportions were analysed using Pearson chi-square statistics which are then converted in to F-statistics to account for survey design.

## Results

### Respondents

The South Australian Health Omnibus Survey samples reflected the South Australian population. In the 2005 survey, for example, 49.0% of respondents were male. Overall, 23.7% were aged 15-29, 27.9% were aged 30-44, 24.0% were aged 45-59 and 24.3% were aged 60+. In 2005, 77.5% of respondents were Australian born (with 3.5% of respondents being Indigenous Australians), 9.4% were from the UK or Ireland; 6.4% were European born and 6.1% were born in other countries. Overall, 6.5% of respondents were still at school, 12.5% did not complete high school, 28.2% had high school education only, 36.3% had completed a trade or certificate and 15.9% had completed a university degree. In 2005, 18.7% of the sample were current smokers and 16.5% (n = 504) were smokers of manufactured cigarettes. In 2006, 20.5% were smokers 17.7% (n = 525) and smoked manufactured cigarettes. In 2007, 19.9% were smokers and 17.2% (n = 414) smoked manufactured cigarettes. In 2008, 19.6% were smokers and 16.4% (n = 464) smoked manufactured cigarettes.

### Awareness of health effects

Table 2 shows the changes in awareness about different health consequences of smoking over time. Top-of-mind responses that smoking caused gangrene increased 6-fold between baseline (2005) and the next year when those warnings were introduced (2006). Awareness that smoking caused mouth cancer more than doubled. Top-of-mind awareness that smoking caused blocked arteries, blindness, stroke, throat cancer and harm to unborn babies all rose significantly after the related warnings were introduced.

Between baseline and 2006 and/or 2007 and/or 2008, significant increases in awareness occurred for nearly all diseases which were also the subject of new pack warnings. No increases were observed in awareness about emphysema, lung cancer, heart disease or addiction, all of which started from a high baseline and/or were already warnings on packs. No increases were observed in health effects unrelated to pack warnings e.g. asthma and impotence.

Unprompted awareness of the Quitline as a service available to help smokers quit rose significantly over time, as did the proportion of smokers able to recite the Quitline number.

### Recall of warnings

Table 3 shows that general recall of anti-tobacco advertising among smoking participants increased markedly in the year that pack warnings were introduced. This effect was specific to cigarette pack warnings, in that while there was a more than doubling in participants reporting (unprompted) they had noticed anti-tobacco information on cigarette packets, virtually no change was observed in relation to television or other sources. Cigarette packets became the second most cited source of anti-tobacco messaging after television. When prompted, 86% of smokers reported noticing new warnings on cigarette packets.

**Table 3:** Noticing warnings and recall of specific pack warnings (unprompted) (smokers of manufactured cigarettes only)

		2005 (n = 504)	2006 (n = 525)	2007 (n = 414)	2008 (n = 464)
**Noticed anti-tobacco advertising in the past 6 months**					
% often or very often		67%	89% ^a^	91% ^a^	93% ^a^
Where - % on TV		89%	93%	91%	93% ^c^
Where - % on radio		19%	22%	20%	24%
Where - % on Internet		<1%	1%	1%	2% ^c^
Where - % on cigarette packs		20%	56% ^a^	57% ^a^	53% ^a^
					
**Notice warnings on cigarette packets (prompted)**					
% Often or very often		63%	86% ^a^	-	-
					
**Recall of new warnings**					
Pictures		0%	14% ^a^	9% ^a^	12% ^a^
Quitline number		<1%	9% ^a^	10% ^a^	12% ^a^
					
Set A	Text/Image				
Smoking causes emphysema	New/Old	3%	**23% **^**a**^	20% ^a^	27% ^a^
Smoking causes mouth and throat cancer	New/New	<1%	**32% **^**a**^	16% ^a,α^	24% ^a^
Smoking causes peripheral vascular disease	New/New	0%	**40% **^**a**^	26% ^a,α^	30% ^a,α^
Smoking clogs your arteries	New/Old	<1%	**11% **^**a**^	7% ^a,χ^	14% ^a,δ^
Smoking - a leading cause of death	New/New	2%	**10% **^**a**^	5% ^c^	10% ^a^
Quitting will improve your health	New/Old	0%	**6% **^**a**^	5% ^a^	7% ^a^
Don't let children breath in your smoke	New/New	1%	**13% **^**a**^	2% ^α^	7% ^c, α, δ^
					
Set B					
Smoking causes blindness	New/Old	<1%		**17% **^**a**^	12% ^a,ϕ^
Smoking doubles your risk of stroke	New/Old	<1%		**9% **^**a**^	5% ^a^
Tobacco smoke is toxic	New/Old	<1%		**4% **^**a**^	4% ^b^
Smoking harms unborn babies*	Old*/New	29%		**31%**	25%
Smoking is addictive^#^	Old/New	8%		**8%**	4% ^c^
Smoking causes heart disease^#^	Old/New	38%		**31% **^**c**^	28% ^b^
Smoking causes lung cancer^#^	Old/Old	56%		**48% **^**c**^	46% ^b^
					
Don't know/can't remember		2%	3%	5% ^c^	5% ^c^

* Very similar to previous warning "smoking in pregnancy may harm the unborn child"

^# ^Old warnings and new warning

^a^Significant difference from baseline (2005) p < 0.001

^b^Significant difference from baseline (2005) p < 0.01

^c^Significant difference from baseline (2005) p < 0.05

^α^Significant difference from 2006 p < 0.001

^χ^Significant difference from 2006 p < 0.05

^δ^Significant difference from 2007 p < 0.001

^ϕ^Significant difference from 2007 p < 0.05

Immediately after the two-phased introduction of the new pack warnings, for all the new health warnings significant increases were observed in the proportion of smokers recalling new messages. There was no increased recall of any of the new graphic warnings that retained old messages. The long standing warning "Smoking causes lung cancer" remained the most recalled (48%), followed by the totally new "Smoking causes peripheral vascular disease" (40%), "Smoking causes throat and mouth cancer" (32%), "Smoking causes heart disease" (31%) and "Smoking harms unborn babies" (31%). Recall was lowest for "Tobacco smoke is toxic" (4%), "Quitting will improve your health" (6%), "Smoking - a leading cause of death" (10%). The graphic new version "Smoking is addictive" remained low at 8%. The mean absolute change for "new"/"new" warnings (i.e. packs with new images and new text; n = 4) was 23% and the mean absolute change for "new"/"old" and "old"/"new" packs (n = 9) was 7%.

### Differences between subgroups

Table 4 shows the different responses of sub-groups to the new warnings. All groups were significantly more likely to report noticing cigarette warnings after the new warnings were introduced.

**Table 4:** Unprompted recall of health warnings by sub-group (smokers of manufactured cigarettes only)

	Notice packs (unprompted)		Gangrene		Emphysema		Mouth & Throat		Arteries		Don't let children		Cause of death		Quitting	
	**2005**	**2006**	**2005**	**2006**	**2005**	**2006**	**2005**	**2006**	**2005**	**2006**	**2005**	**2006**	**2005**	**2006**	**2005**	**2006**

Age groups																
15-29 years (n_2005 _= 135; n_2006 _= 159)	26.1	60.7^α^	0.0	39.5^α^	2.2	19.8^α^	1.7	36.5^α^	0.0	7.6^χ^	0.7	18.4^α^	2.2	12.2^β^	0.0	2.6
30-44 (n_2005 _= 205; n_2006 _= 168)	15.4	56.5^α^	0.5	46.5^α^	3.4	29.4^α^	0.3	31.2^α^	0.5	11.7^α^	1.2	12.6^α^	1.4	8.0^χ^	0.0	7.0^β^
45-59 (n_2005 _= 109; n_2006 _= 130)	20.5	52.8^α^	0.4	37.0^α^	5.8	21.8^α^	1.6	28.3^α^	0.0	13.6^α^	0.9	8.4^α^	2.1	10.1^χ^	0.4	9.0^α^
60+ (n_2005 _= 55; n_2006 _= 68)	25.7	46.9^χ^	0.0	30.2^α^	1.6	18.7^α^	0.0	28.8^α^	0.9	15.4^β^	0.0	7.4	0.0	5.7 ^χ^	0.0	4.2
Sex				b				b				b	c	c		
Male (n_2005 _= 258; n_2006 _= 284)	18.9	52.5^α^	0.2	33.4^α^	2.9	24.9^α^	0.8	24.1^α^	0.4	8.5^α^	0.8	8.0^α^	2.6	6.5	0.2	4.4^α^
Female (n_2005 _= 246; n_2006 _= 241)	22.1	59.3^α^	0.4	47.6^α^	4.0	21.2^α^	0.9	40.8^α^	0.2	14.8^α^	1.0	18.1^α^	0.6	13.0^α^	0.0	7.5^α^
Planning to quit in next 6 months																
Yes (n_2005 _= 275; n_2006 _= 287)	18.9	54.7^α^	0.0	38.8^α^	3.2	21.6^α^	0.6	34.5^α^	0.4	9.2^α^	1.4	14.0^α^	1.6	8.5^β^	0.0	6.0^α^
No/Can't say (n_2005 _= 229; n_2006 _= 235)	22.3	56.7^α^	0.6	41.3^α^	3.7	25.2^α^	1.3	28.5^α^	0.2	14.1^α^	0.3	11.0^α^	1.6	10.7^α^	0.2	5.6^α^
TOTAL (n_2005 _= 504; n_2006 _= 525)	20.5	55.6^α^	0.3	40.0^α^	3.4	23.2^α^	0.9	31.8^α^	0.3	11.4^α^	0.9	12.7^α^	1.6	9.5^α^	0.1	5.8^α^

	**Correct recall Quitline no**.		**Unborn babies**		**Blindness**		**Lung cancer**		**Heart disease**		**Stroke**		**Addictive**		**Toxic**	

	**2005**	**2007**	**2005**	**2007**	**2005**	**2007**	**2005**	**2007**	**2005**	**2007**	**2005**	**2007**	**2005**	**2007**	**2005**	**2007**
Age groups		d		d			b						c		c	
15-29 years (n_2005 _= 135; n_2007 _= 119)	7.2	26.8^β^	27.4	41.3	0.9	11.9^α^	59.3	50.2	45.9	28.7^χ^	0.7	5.4^χ^	12.9	11.6	1.9	4.1
30-44 (n_2005 _= 205; n_2007 _= 148)	5.8	11.9	33.6	34.2	0.2	19.9^α^	62.6	56.0	36.9	38.0	0.7	10.5^α^	8.9	8.3	0.0	5.6^α^
45-59 (n_2005 _= 109; n_2007 _= 109)	1.0	6.8^β^	25.6	22.4	0.0	20.3^α^	51.6	41.0	35.0	26.9	0.7	10.7^β^	2.6	4.2	0.0	3.5^χ^
60+ (n_2005 _= 55; n_2007 _= 39)	0.0	1.8	18.3	13.0	0.0	13.1^β^	33.2	38.3	26.7	22.0	0.0	7.5^χ^	5.4	8.3	0.0	3.6
Sex																
Male (n_2005 _= 258; n_2007 _= 200)	5.9	10.7	29.4	28.0	0.2	14.5^α^	58.1	45.1^χ^	39.1	29.2	0.9	8.3^α^	6.9	7.9	0.0	4.4^β^
Female (n_2005 _= 246; n_2007 _= 214)	3.6	16.8^α^	27.6	34.1	0.5	19.5^α^	54.1	52.1	36.4	32.6	0.3	9.3^α^	9.6	8.5	1.0	4.5^χ^
Planning to quit in next 6 months																
Yes (n_2005 _= 275; n_2007 _= 209)	5.9	12.9^χ^	27.9	35.8	0.2	18.3^α^	59.2	51.2	41.0	32.8	0.5	9.8^α^	7.6	8.0	0.3	3.7^β^
No/Can't say (n_2005 _= 229; n_2007 _= 205)	2.8	14.8^β^	29.3	26.4	0.5	15.8^α^	52.5	46.2	33.9	29.0	0.8	7.8^α^	9.0	8.4	0.8	5.1^χ^
TOTAL (n_2005 _= 504; n_2007 _= 414)	4.5	13.9^β^	28.5	31.1	0.3	17.1^α^	56.1	48.7^α^	37.8	30.9^α^	0.6	8.8^α^	8.2	8.2	0.5	4.4^α^

Significant difference between years: Chi-square: α = p < 0.001; β = p < 0.01; χ = p < 0.05

Significant differences within year (between subgroups): Chi-square: a = p < 0.001; b = p < 0.01; c = p < 0.05; Chi-square for trend: d = p < 0.001

"Smoking harms unborn babies" was more recalled by younger smokers. Female smokers were more likely to recall warnings relating to gangrene, mouth cancer and children than their male counterparts. These were the exception; more often than not, there were no significant differences in recall of the warnings between sub-groups. Generally, warnings with the highest increased recall overall (e.g. "Gangrene" and "mouth and throat cancer"), were also the warnings with the highest increases in recall among all sub-groups. Generally, warnings that had weaker recall overall were also the weakest within the sub-groups.

Younger smokers were significantly better able to recount the Quitline number than older smokers after it was introduced onto cigarette packets, showing a dramatic increase from baseline. After the new warnings were introduced, awareness of the Quitline number increased in both smokers interested to quit in the next 6 months and those not interested. A greater gain was observed among smokers not (yet) seriously considering quitting.

### Effects over time

Tables 2 and 3 present data from 2005 to 2008. Data in Table 3 show that cigarette packets remained a noticed source of anti-tobacco advertising. Table 3 shows indications of decline in recall of warnings introduced in early 2006 (Set A) during 2007, with some recall rebounding again in 2008. Similarly, some fall off of Set B warnings recall occurred in 2008. Table 2 shows very little evidence of decline in recall of health effects of smoking specific to new packet warnings, 2 years post first implementation. Table 3 shows that two-years post implementation, "Smoking causes lung cancer" remained the highest recalled pack (46%), followed by a second tier: "peripheral vascular disease", "heart disease", "emphysema", "unborn babies", "mouth and throat cancer" ranging from 30% to 24% unprompted recall. Those with lowest impact initially remained low, with recall ranging down to 4% for "toxic" and "addictive".

## Discussion

This study demonstrates that new graphic cigarette packet warnings coincided with increased awareness among smokers of the health consequences of smoking observed in cross-sectional surveys of South Australian smokers across four years. While it is possible that these increases in awareness of smoking related illnesses may have happened due to other influences or by chance, new graphic cigarette packet warnings are the most likely cause of the increases in awareness of smoking related disease.

Over the time that new graphic cigarette pack warnings were introduced, we observed substantial increases in top-of-mind awareness of diseases that were the subject of new warnings, and no increases in awareness of other health effects. Further supporting evidence was provided by the increased proportion of smokers who reported noticing warnings on cigarette packets after the new warnings were introduced. We also observed significant increases in smokers' unprompted recall of pack warnings as a source of anti-tobacco information. Again, this effect was isolated to pack warnings and not generalised to other sources such as television. After the new warnings were introduced, cigarette packets became second only to television as a recalled source of anti-tobacco messages for smokers. Arguably, noticing anti-tobacco messages on television could be at saturation point, after 20 years of regular anti-smoking campaigns. However, there was no increase in noticing messages on the less used media of radio or on the internet.

As observed in this study, it has been demonstrated previously that new messages delivered via television campaigns can markedly increase awareness of smoking related diseases in a 6-month period. As was the case in this study, the effects on awareness were specific to the diseases highlighted in the advertisements and not generalised to all smoking related illnesses [21].

Similar to the Canadian experience [14], cigarette packets became a prominent important source of anti-tobacco information, after graphic cigarette warnings were introduced, and they remained so in the 2 years after they were introduced. In this study there was some evidence of a spike of recall of new warnings with some short term attrition, followed by more steady results. Importantly, most of the data in this study are on unprompted recall, so it is to be expected that top-of-mind recall of warnings and associated health beliefs would be highest in the year that new warnings are introduced, and that it might subsequently be displaced from top-of-mind by more recent warnings. This would be consistent with Fishbein & Ajzen's [5] contention that people's salient beliefs about the consequences of any contemplated action do not exceed 5-9 in number. Warning and health effect recall does appear to stabilise but more longitudinal data are required to ascertain longer term effects.

The impact varied greatly between warnings. "Smoking causes heart disease" and "lung cancer" are warnings that have been on Australian cigarette packets for a long time (as text-based warnings). They are also diseases which a high proportion of smokers were already aware were caused by smoking, at baseline. Awareness of these diseases and recall of these pack warnings remained high but demonstrated no improvement once the new graphic warnings were introduced. "Smoking is addictive" was also a graphic adaptation of an old text-based warning. Like "heart disease" and "lung cancer" messages, no significant increase was observed in awareness of the relationship with smoking, or in recall of the warnings. However, unlike "heart disease" and "lung cancer", "addictive" stayed at a low level on both measures. At baseline, smokers already had a high awareness of the relationship between smoking and emphysema. The introduction of the completely new "emphysema" warning (with a familiar graphic - see Table 1), did increase recall of the warnings but did not shift the already high awareness of the disease among smokers. These four cases suggest that adding a graphic image (or at least these graphic images) to an old warning or an "old news" disease did nothing to improve awareness or recall.

By contrast, when baseline awareness of a disease/damage caused by smoking was low, and the disease/damage had not previously been used as a pack warning, awareness grew very significantly. Greatest growth in awareness was observed in relation to gangrene (4% to 27%) and mouth cancer (10% to 24%), both of which were "new news" and contained new images. Even topics that involved new warnings but images and messages which had been the subject of previous tobacco control campaigns (see Table 1) induced significant growth in awareness: "blocked arteries" increased 8%; "blindness" increased 9%; and "stroke" increased 8%. Hence, based on these examples, adding a new graphic image (or at least these graphic images) to a new warning would seem to improve awareness considerably, as does adding a familiar graphic image to a new warning.

Moreover, those warnings most recalled across the board and in different subgroups were those which were "new news", and used new images and particularly images of body parts likely to elicit a visceral "yuck" response. "Gangrene" (40% unprompted recall) and "mouth cancer" (32%) were dominant in this sense. Although "heart disease", "lung cancer" and "harms unborn babies" were also recalled well (49%, 31% and 31% respectively) and contained visceral or emotive images, the new packs failed to provoke an improvement in recall over baseline, suggesting their high recall cannot be attributed to the graphic imagery.

The warnings with weakest recall were "Tobacco smoke is toxic" (4%), "Smoking is addictive" (8%), "Quitting will improve your health" (6%), "Smoking - a leading cause of death" (11%). With the exception of "Smoking is addictive" all of these warnings are general rather than specific about the consequences of smoking and none of these warnings contain images of body parts. Anti-tobacco television campaigns have consistently demonstrated that images and messages eliciting a visceral response and messages that are novel or "new news" are more likely to be attended to and have impact on quitting behaviour [20,22,30]. This study demonstrates that these findings are generalisable to cigarette pack warnings. This study also demonstrates that these findings apply to smokers in general, as well as to different subgroups of smokers.

The addition of the Quitline number to the cigarette packet appears to have increased general top-of-mind awareness of the availability of the Quitline service. This is noteworthy because the Australian Quitline has been operating for over two decades and already enjoyed high levels of awareness. Although not significant, a coincident trend was observed in increased use of the Quitline as a source of help to quit. The proportion of smokers who knew the Quitline number doubled; and in 2007, one in eight smokers could recite the number accurately. An independent study demonstrated that calls to the Australian Quitline doubled in the year after the new warnings were introduced [31].

Health promotion often aims to segment different messages for different markets in the expectation of having greater impact. The case has been made, using mass-media quit campaigns as the example, that this is unnecessary and even counter-productive because it comes at a cost, namely the dilution of resources required for population-wide campaigns [32]. Comparisons between warnings as well as comparisons between population sub-groups show that what "works", works well across the board and what "doesn't work" across the board, also doesn't work well with any subgroup. The only exception in this study was the greater propensity shown by women and younger smokers to respond to warnings about unborn babies and children, presumably because their closer specific personal relevance. Overall, this study provides another example of a population-based intervention working well with both smokers generally and within subgroups, building the case for non-segmented interventions.

This study provides clear evidence that Australia's new graphic cigarette packets succeeded in attracting the attention of Australian smokers. A limitation of this study is that it did not explicitly ask smokers what, about the different warnings, attracted their attention, nor did it ask smokers directly about their perceptions of the credibility of different warnings. Some warnings may have been better recalled than others because smokers thought they made outrageous and incredible claims. However, this study provides evidence that smokers did find the highly recalled warnings credible. The fact that smokers' unprompted recall of illnesses caused by smoking increased in line with the increased recall of warnings is evidence of this. Changes in awareness about the harms of smoking are an important antecedent to behaviour change for many smokers. Whether behaviour change did follow was not measured in the current study.

This study provides support for the Framework Convention on Tobacco Control Article 11, mandating large cigarette packet warnings and recommending graphic imagery. Tobacco control policies, such as the FCTC and Australia's National Tobacco Control Strategies, recognise the complexity of smoking behaviour and the multiple behavioural and structural interventions required to reduce tobacco's toll. Graphic cigarette packet warnings play a role as one component of a comprehensive suite of tobacco control interventions.

## Conclusions

In conclusion, Australian graphic cigarette packet warnings have been shown in this study to have caught the attention of Australian smokers who have extended the range of their beliefs about the harmful consequences of smoking. Lessons for policy makers planning to introduce graphic warnings are that, as with anti-tobacco television campaigns, "new news" attracts more attention than "old news" and visceral images are more powerful than other graphics. The importance of "new news" should also be considered by policy makers in countries where graphic warnings have already been introduced, as many of the health effects of smoking are unfamiliar to many smokers and an opportunity exists to increase awareness by updating and rotating warnings.

## Competing interests

The authors declare that they have no competing interests.

## Authors' contributions

All authors participated in the conceptual development, the study design, the writing and editing of the article. In addition, CLM was also responsible for data analysis and drafting of the manuscript. All authors read and approved the final manuscript.

## Pre-publication history

The pre-publication history for this paper can be accessed here:

http://www.biomedcentral.com/1471-2458/11/238/prepub
